# Enteric *Escherichia coli* O157:H7 in Cattle, and the Use of Mice as a Model to Elucidate Key Aspects of the Host-Pathogen-Microbiota Interaction: A Review

**DOI:** 10.3389/fvets.2022.937866

**Published:** 2022-07-11

**Authors:** Maximo E. Lange, Richard R. E. Uwiera, G. Douglas Inglis

**Affiliations:** ^1^Agriculture and Agri-Food Canada, Lethbridge Research and Development Centre, Lethbridge, AB, Canada; ^2^Department of Agricultural, Food and Nutritional Science, University of Alberta, Edmonton, AB, Canada

**Keywords:** *Escherichia coli* O157:H7, cattle, intestinal colonization, immune response, competitive exclusion, mouse models, stress, corticosterone

## Abstract

Enterohemorrhagic *Escherichia coli* (EHEC) serotype O157:H7 is responsible for foodborne disease outbreaks, typically associated with the consumption of undercooked foods contaminated with cattle manure containing the bacterium. At present, effective mitigations do not exist. Many of the factors regulating enteric colonization by *E. coli* O157:H7 in cattle, and how cattle respond to the bacterium are unknown. In this regard, intestinal colonization locations, shedding patterns, interactions with the enteric microbiota, and host immune responses to infection are current knowledge gaps. As disturbances to host homeostasis are believed to play an important role in the enteric survival of the bacterium, it is important to consider the potential importance of stress during cattle production. Husbandry logistics, cost, and the high genetic, physiological, and microbial heterogeneity in cattle has greatly hampered the ability of researchers to elucidate key aspects of the host-pathogen-microbiota interaction. Although mice have not been extensively used as a cattle model, the utilization of murine models has the potential to identify mechanisms to facilitate hypothesis formulation and efficacy testing in cattle. Murine models have been effectively used to mechanistically examine colonization of the intestine, host responses to infection, and to interactively ascertain how host physiological status (e.g., due to physiological stress) and the enteric microbiota influences colonization and disease. In addition to reviewing the relevant literature on intestinal colonization and pathogenesis, including existing knowledge gaps, the review provides information on how murine models can be used to elucidate mechanisms toward the development of rationale-based mitigations for *E. coli* O157:H7 in cattle.

## Introduction

*Escherichia coli* is commonly found in the intestinal tract of mammals, and is the most abundant facultative anaerobe within the human intestinal tract (1, 2). Certain serotypes of *E. coli* can develop a mutualistic relationship with the host, while other serotypes are pathogens or opportunistic bacteria that incite acute intestinal and extra-intestinal disease, respectively. The serotypes of *E. coli* are defined by the combination of their surface O (somatic), H (flagellar), and sometimes K (capsular) antigens (1). These surface antigens are important determinants for inducing disease within the host. In many countries, *E. coli* O157:H7 is of particular public health interest as a consequence of its pathogenicity in human beings, where it can incite non-bloody diarrhea, hemorrhagic colitis, and potentially, hemolytic uremic syndrome (HUS) (2). As such, *E. coli* O157:H7 is considered to be an important zoonotic pathogen, and its main reservoirs are healthy domesticated ruminants, predominantly cattle, and to a lesser extent sheep and goats (3). A multitude of mitigation approaches have been evaluated in ruminants (4, 5). However, no single method to date has been successful in eliminating *E. coli* O157:H7 from the intestine of ruminants. In comparison to efficacy assessments, relatively few studies have focused on replicating and understanding natural colonization patterns, immune responses, and potential symptomatic infections (6–12). It is anticipated that addressing current knowledge gaps will facilitate the development of effective strategies to reduce enteric colonization by *E. coli* O157:H7. For example, gaining an understanding of key aspects of the host-pathogen-microbiota interaction may lead to effective competitive exclusion strategies (13). Host stress is believed to be an important aspect influencing the host-pathogen-microbiota. Stress is recognized as a factor that can affect host physiological processes and homeostasis, immune responses, the environment of the gastro-intestinal tract (GIT), and inter-bacterial communication (14). Indeed, stress hormones can potentiate virulence mechanisms of *E. coli* O157:H7 directly influencing its colonization (15). A significant challenge facing researchers is the difficulty of conducting mechanistic research in cattle due to significant challenges in animal husbandry, cost, and prominent genetic, physiological, and microbial heterogeneity in cattle. The use of model animals may facilitate the identification of mechanisms (e.g., factors affecting GIT colonization, infection, and competitive exclusion). Mice have been used as a model organism to study various aspects of the *E. coli* O157:H7-host interaction, but primarily from a human health perspective (16–21). The current review summarizes key aspects of *E. coli* O157:H7 and its interaction with ruminant hosts, including knowledge gaps. Additionally, the review examines the utility of using mice as a model for ruminants, and how mice may be applied to address current knowledge gaps and facilitate the rationale-based identification and evaluation of mitigations.

## Pathogenesis in Human Beings

*Escherichia coli* O157:H7 is an enterohemorrhagic *E. coli* (EHEC) belonging to a group of bacterial strains that are capable of expressing Shiga toxin (Stx), that cause hemorrhagic colitis and HUS, characterized by developing attaching/effacing (A/E) lesions on epithelial cells (1). As such, *E. coli* O157:H7 is only one of multiple serotypes that belong to the specific EHEC pathotype. This group of EHEC is included in a larger cohort of *E. coli* bacteria, known as Shiga toxin *E. coli* (STEC) or verotoxigenic *E. coli* (VTEC), all of which have the characteristic ability of producing Shiga toxin. All EHEC are believed to be pathogens, whereas not all STEC or VTEC bacteria are pathogenic (1).

*Escherichia coli* O157:H7 was first recognized as an incitant of enteric disease in human beings in 1982 (22). Since then, the bacterium has been linked to diverse foodborne disease outbreaks (2). Notably, *E. coli* O157:H7 can be highly virulent, as a relatively low infective dose of *E. coli* O157:H7 is required to induce disease, and it has been shown that ingestion of as few as 100 EHEC cells can cause infection (2).

**Figure 1:**
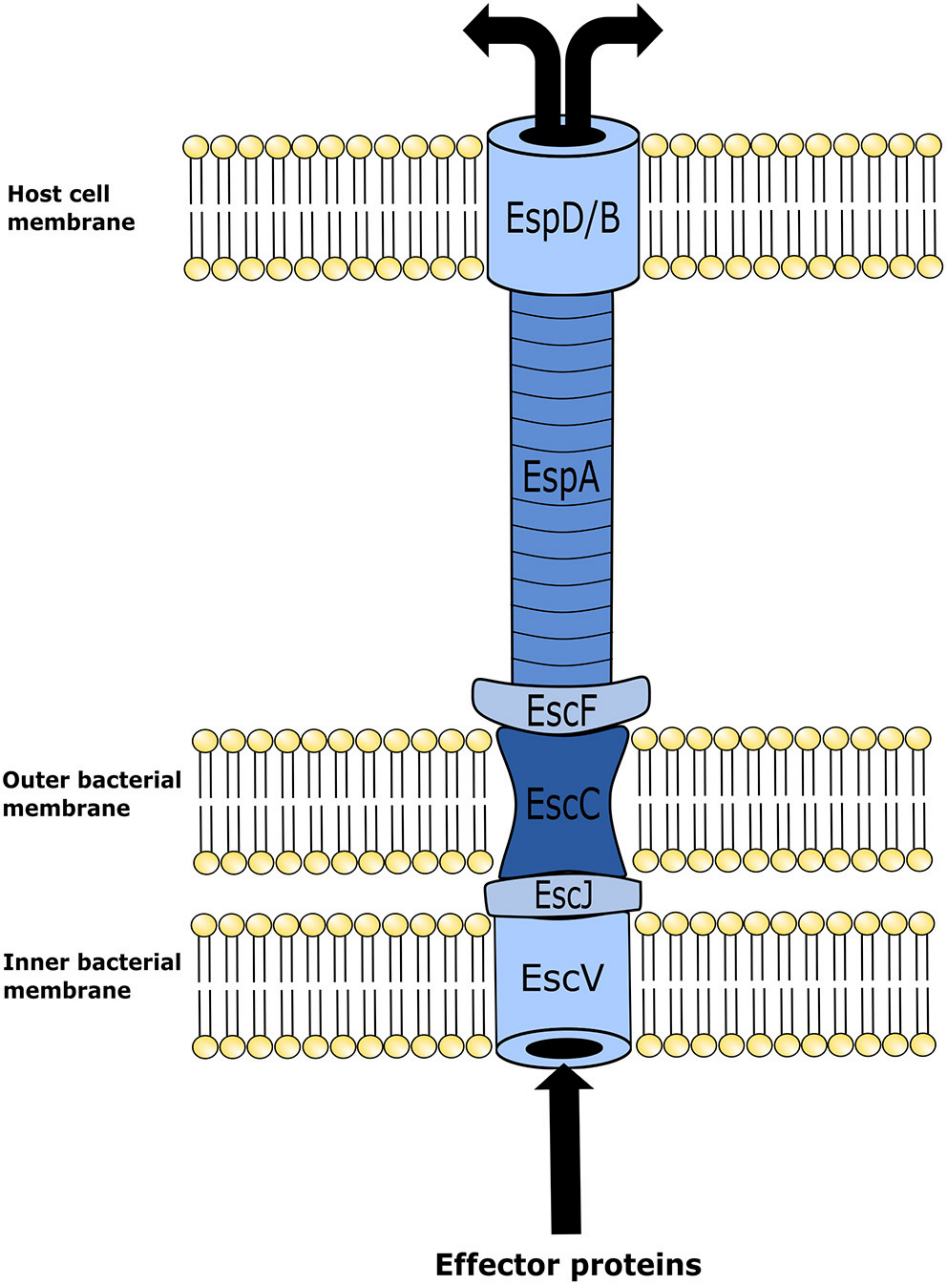
Illustration of enterohemorrhagic Escherichia coli (EHEC) type three secretion system structure. This mechanism is utilized by EHEC to introduce effector proteins into the host cell. Upon locus of enterocyte effacement (LEE) activation, the proteins EscV and EscC establish themselves in the inner and outer membranes of the bacterium, respectively, forming an annular complex. The structural lipoprotein EscJ is then placed within the periplasmic space between EscV and EscC. In conjunction, these proteins make a corridor on the bacterial membrane for effector proteins to be transported out of the cell. In turn effector proteins are moved from the bacterial membrane into the host cell using a needle-like structure that is elongated from the bacterial membrane structure to an annular complex formed in the host cell membrane. This needle-like structure is comprised of the *E. coli* secreted proteins, EscF and EspA. EspA forms bonds with EscF, and polymerizes into the hollow needle-like structure that extends out and allows the connection with the host cells of the intestinal epithelium. In combination with EspA, protein EspB and EspD form a pore on the cell membrane of the host epithelial cell. Effector proteins then travel through the fully formed secretion system from the bacterium into the host cell.

Although EHEC cellular mechanisms for inducing disease in humans are highly complex, for the scope of this review only a brief description will be provided as background information. The attachment between the bacterium and intestinal epithelial cells occurs by means of A/E lesions. Genes with the capacity of encoding proteins required for A/E are found in the locus of enterocyte effacement (LEE) pathogenicity island (PAI) (23). Upon activation, LEE operons encode for proteins involved in a type-III secretion system (TTSS); multiple effector proteins are required for binding of the bacterium to epithelial cells, formation of A/E lesions, and disruption of epithelial cell function. The TTSS is required to insert various effector proteins into the host cell. It is an apparatus that forms in both the inner and outer membranes of bacteria ultimately forming a “needle” that extends from the bacterial cell and contacts the host cell (24) (Figure 1). Once the TTSS is established, a translocated intimin receptor (Tir) is delivered through it and localizes within the host epithelial cell membrane, and acts as the receptor for an adhesin protein (intimin) on the membrane of EHEC. This Tir/intimin connection enables the attachment of EHEC to host cells (23). Tir also binds to the host cytoskeleton and induces polymerization of actin with the final formation of actin rich pedestals under the bacterium. Following formation of the pedestal, the epithelial microvilli are effaced inducing cell injury and intestinal inflammation within the distal GIT (1, 23).

A prominent virulence factor of EHEC is Stx, and this protein is considered the primary causative factor for inducing HUS. Importantly, not every EHEC infection/case will develop into HUS. HUS is a potentially fatal clinical manifestation of disease in human beings characterized by acute renal failure, thrombocytopenia, and microangiopathic hemolytic anemia. Shiga toxin is phage encoded in the bacterial genome, and it is only released in the presence of disturbances to the bacterial DNA, cell wall or protein synthesis. Different antibiotics can affect bacterial DNA, membrane, or protein synthesis. This is the primary reason why antibiotic therapy to treat EHEC in people is still a topic of debate given that antibiotics can potentially cause the release of Stx from the bacterial cell (25). This toxin is comprised of two serologically distinct serotypes (i.e., Stx1 and Stx2); and these virulence factors can be expressed individually or together. The toxin has a B subunit that binds to the receptor glycolipid globotriaosylceramide (Gb3) on the cell surface, while the A subunit is introduced into the cell where it acts on the 60S subunit of the ribosome cleaving a single adenine from the 28S rRNA, and thus inhibiting cellular protein synthesis and subsequently leading to host cell death (1). Human cells such as Paneth cells, endothelial cells, and kidney epithelial cells possessing the Gb3 receptor are susceptible to Stx binding and cell injury. In contrast, cattle lack the expression of Gb3 receptors in kidney glomeruli, and it is believed that this is why bovids do not develop HUS (26).

## Ruminant Reservoirs

The intestinal tract of bovine species, and small ruminants such as sheep and goats, is the primary animal reservoir for human infectious *E. coli* O157:H7. The mechanisms involved in the anatomical localization, intestinal colonization, shedding patterns, and low prevalence of *E. coli* O157:H7 disease in ruminants are not fully understood. Importantly, the elucidation of these mechanisms may facilitate the development of effective on farm mitigation strategies to reduce the transmission of *E. coli* O157:H7 from livestock to people. Contamination of the environment with the bacterium can be quite variable. Shedding the bacterial cells in feces can be intermittent, the duration of shedding events can be short lived, and the quantity of cells released into the environment vary from 10^1^ to 10^9^ colony forming units (CFU)/g of feces (27). Typically, cattle shed EHEC at high densities for short periods of time, followed by extended periods in which no fecal shedding occurs or the bacterium is shed at low cell densities (28). Moreover, the prevalence of EHEC shedding in feces tends to be higher during the summer and autumn, and lower during the winter months (29). Shedding of the bacterium among different cattle can also be variable. As an example, cattle that shed the bacterium at densities greater than 10^4^ CFU/g in feces are considered super shedders (27). Omisakin et al. (30) found that the 9% of cattle shedding at higher than 10^4^ CFU/g in feces corresponded with more than 96% of the total *E. coli* O157:H7 present in the cattle tested. This supports the possibility that only a small group of animals account for the majority of the *E. coli* O157:H7 released in the farm environment. The duration of the super shedding periods still remains unknown, and not all feces collected from shedding cattle are positive for *E. coli* O157:H7, supporting the intermittent shedding of the bacterium among different cattle (27).

## Intestinal Colonization in Cattle

The intestinal location and conditions for colonization of EHEC in cattle is currently a subject of scientific debate. In cattle, *E. coli* O157:H7 is mainly identified with the large intestine, including the cecum, and proximal and distal colon, and can be closely associated with the mucosal epithelium of the rectum (9, 31). Indeed its been demonstrated that after inoculation of ruminants with *E. coli* O157:H7, the bacterium does not tend to persist in the rumen, upper or mid small intestine, but will colonize in both the distal ileum, cecum, and colon, with the greatest quantities of bacteria isolated within the large intestine (31). Interestingly, the highest densities of cells are isolated from feces, even after the bacterium is no longer present in intestinal tissues (29). It is noteworthy that densities of *E. coli* O157:H7 on the surface of feces are higher than within the fecal core, suggesting that the bacterium is prevalent in the distal parts of the large intestine (9). In addition, several studies have indicated that the terminal rectum, a region rich in lymphoid tissue, is the primary site of colonization for *E. coli* O157:H7 (9), as the bacterium is most commonly isolated from this region of the GIT. It is believed that *E. coli* O157:H7 establishes intestinal adherence at the distal rectum. However, it is important to note that this research has primarily examined cattle that have been inoculated with *E. coli* O157:H7 and not for natural infections. Collectively, this information indicates that the niches and mechanisms required for *E. coli* O157:H7 colonization and survival within the intestinal tract of cattle have yet to be fully determined.

## Clinical Features and Susceptibility in Cattle

Ruminants, particularly mature cattle, act as an asymptomatic reservoir for EHEC. However, it has been demonstrated that newborn calves (<36-h-old) can present clinical manifestation and tissue injury associated with *E. coli* O157:H7 infections; this includes watery diarrhea, neutrophilic infiltration of the intestinal mucosa, necrosis and sloughing of epithelial cells and A/E lesions in the large and small intestines (32). In 70-day-old calves, inoculation with *E. coli* O157:H7 caused disruption of the epithelium in the ileum, atrophic villi, and neutrophilic exudation into the lumen (7). Furthermore, the colon also presented mucosal necrosis and mesenteric lymphadenopathy 7 days post-inoculation. Interestingly, prominent cellular sloughing and neutrophilic micro-abscesses were found in the recto-anal junction (7). Finally, there is a higher prevalence of EHEC in cattle following long distance transportation, changes in diet, and antibiotic therapy, possibly linked to immunocompetence of the animals and/or to disturbances to the structure of the intestinal tract microbiota (28).

## Bovine Immune Responses to Enteric Colonization

Although cattle are the main reservoir of *E. coli* O157:H7, infected adult cattle typically do not present overt clinical manifestation of disease. However, *E. coli* O157:H7 is an established commensal organism of cattle and could be potentially pathogenic. There is convincing evidence of a protective immune response by the bovine host in response to EHEC colonization (10). In this regard, EHEC have been found to form attaching effacing lesions on the intestinal epithelium; however, in order to establish this close attachment with the host mucosa, the bacterium must first contact the epithelium. For instance, interaction between flagella H7 and enterocytes leads to the initiation of the TTSS (10). The flagellum is recognized by toll-like receptor 5 (TLR-5) of the host that results in the activation of nuclear factor (NF)-κβ, and subsequently production of interleukin 1B (IL-1B), interleukin 8 (IL-8), and tumor necrosis factor alpha (TNFα) (10). The presence of EHEC lipopolysaccharide (LPS) also activates toll-like receptor 4 (TLR-4), which stimulates a similar pro-inflammatory response (10). Once the TTSS is activated, the bacterium attaches to the enterocyte and this process is associated with only mild granulocytic mucosal infiltration accompanied by modest exfoliation of the epithelium at sites of colonization (8). In contrast, neonatal calves challenged with *E. coli* O157:H7, present marked lamina proprial granulocytic infiltration within the large intestine accompanied by substantive tissue congestion, edema and epithelial degeneration (32). Disease severity is reduced in calves greater than 3-weeks-of-age, suggesting that the pathogenicity of *E. coli* O157:H7 in cattle is age dependant (32).

Corbishley et al. (6) studied gene expression of the rectal mucosa in 12-week-old calves inoculated with *E. coli* O157:H7. Cytokine profiles directed toward a T helper 1 (Th1) response were observed, with an increase in the expression of interferon gamma (IFNγ) and T-bet as compared to control animals. There was also a reduction in transforming growth factor beta (TGFβ) expression in inoculated animals while maintaining elevated IFNγ levels providing further evidence of an ongoing pro-inflammatory Th1 response (6). Additionally, there were no observed changes in cytokines related with a Th2 response. In 70-day-old calves, infection with *E. coli* O157:H7 increased transcription of tracheal antimicrobial peptide (TAP) in the ileum as well as transcription of lingual antimicrobial peptide (LAP) in the recto-anal junction (7). Furthermore, it was observed that *E. coli* O157:H7 augmented the production of mucin in the recto-anal junction after 14 days of infection (7). Increases of mucin production suggest an epithelial response toward eliminating the bacterial pathogen.

The presence of neutralizing antibodies against virulence factors of *E. coli* O157:H7 in naturally infected cattle have been observed. More specifically, antibodies generated against Stx1 and Stx2, LPS, TTSS proteins, intimin, tir, EspA, EspB, and H7 flagellin (10). Moreover, evidence suggested that Stx can supress immune cell activity in cattle. In this regard, peripheral blood mononuclear cells isolated from calves previously administered Stx2-positive *E. coli* O157:H7 failed to generate proliferative responses following a challenge with heat killed Stx2-positive *E. coli* O157:H7 *in vitro*. This differed from peripheral blood mononuclear cells isolated from animals that were administered Stx-negative *E. coli* O157:H7 bacterial cells and developed a robust response when re-challenged with heat killed Stx2-positive *E. coli* O157:H7 *in vitro* (33). Studies examining the transcriptome of the recto-anal junction in naturally infected super shedders compared to non-shedders, showed a down regulation of multiple immune factors in super shedders. This reduction was mainly related with the function and chemo-attraction of B-cells, and the migration of neutrophils, macrophages, and dendritic cells (11). This suggests a potential decrease in numbers of granulocytes in areas of colonization of *E. coli* O157:H7, which facilitates the establishment of the bacterium within the GIT. It is still unclear if the reduced protective immune responses are associated with the colonization ability of individual bacterial strains or due to a differing susceptibility of super shedding hosts.

## Control in Cattle

Multiple approaches have been implemented to reduce or eliminate the presence of EHEC in cattle or within cattle processing plants. Methods to reduce the amounts of shedding and presence of the bacteria within the GIT before the animal arrives at the abattoir are known as pre-harvest measures. Pre-harvest prevention is implemented to address high levels of bacterial contamination of cattle hides, particularly during the summer months, as elevated EHEC cell densities can overwhelm the sanitary measures utilized in plants to control EHEC. Many strategies implemented to reduce bacterial load target key farm production practices that are thought to affect proliferation of EHEC in cattle. These strategies target changes in concentrations of grain within the diet, the addition of prophylactic and therapeutic amounts of antimicrobial agents to feed, changes in intensity and density of cattle production, and methods of manure disposal (4). Many of these adjustments, however, are either partially effective or completely ineffective (4). As a result, the development of alternate strategies to reduce the level of EHEC carriage and shedding are active areas of investigation.

Many pre-harvest control methods aim to limit exposure of animals to EHEC by reducing animal density, exposure to wildlife potentially carrying the bacteria, and enhancing feed hygiene; however, most often these practices are impractical for Canadian and international cattle production systems (5). Other mitigation strategies focus on reducing the amount of pathogen within the GIT by utilizing feed that can alter short chain fatty acid concentrations, reducing pH, and altering the composition of resident intestinal bacteria (5). Furthermore, probiotics such as *Lactobacillus acidophilus* are commercially available, and have been reported to help reduce the shedding of EHEC in feces (34). Finally, strategies that directly target EHEC such as hide washing, administration of antibacterial agents, such as sodium chlorate, to feed and water, and the use of bacteriophages have been evaluated with variable results (5, 35). Several vaccines against *E. coli* O157:H7 have been developed and there are currently two commercial vaccines available. The first vaccine is directed at enhancing mucosal immunity against the TTSS, thereby reducing or preventing bacterial adherence (4). The second vaccine stimulates the generation of antibodies against a siderophore receptor. This receptor is needed to sequester iron and antibodies binding to the receptor prevents *E. coli* O157:H7 from up-taking iron, an essential function for bacterial survival (4). Vaccination has proven only partially effective in reducing prevalence of the bacteria within cattle, and has been unable to eliminate the bacteria from an entire cattle herd. Although some of the currently available pre-harvest controls are partially effective, it is plausible that a combination of multiple methods may increase their efficacy. However, no practical strategy that is economically feasible to reduce EHEC has been developed as of yet. Thus, the incidence of human infection with *E. coli* O157:H7 has remained steady globally and throughout the years (4).

## Stress and Glucocorticoid Effects in Cattle

Periods of disturbance to homeostasis are inevitable during livestock production. Social mixing, animal restraint and handling, introduction of cattle to new environments, transportation, weaning and management (castration, vaccination, dehorning and branding) are examples of cattle production activities that induce stress.

Stress has been studied extensively and it is defined herein as the sum of all biological reactions to physical, emotional, or mental stimuli that disturb homeostasis (36). This threat is referred to as the stressor.

**Figure 2:**
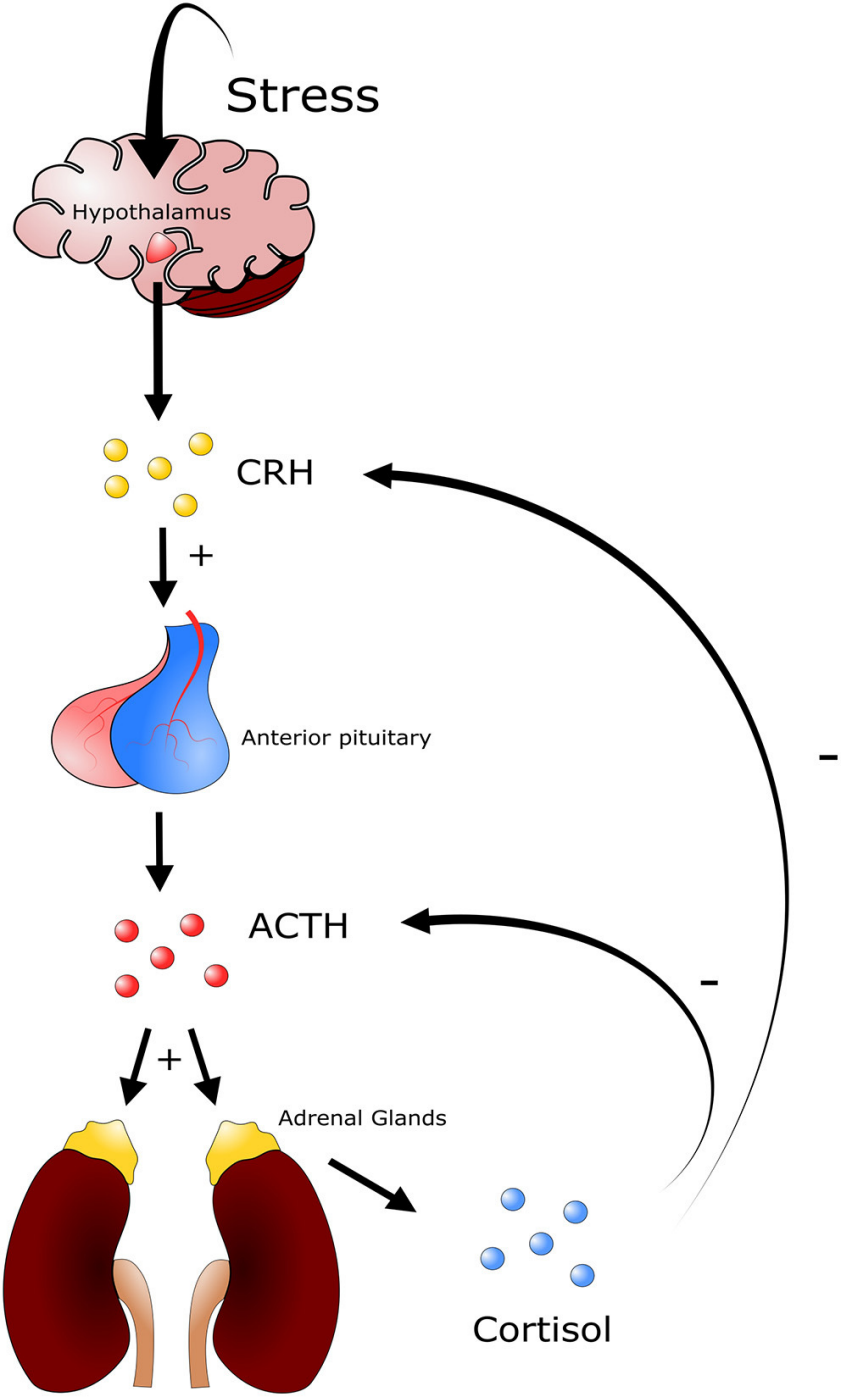
Depiction of the hypothalamic pituitary adrenal axis. Different stressors will induce a response from the hypothalamus triggering the release of corticotrophin releasing-hormone (CRH). CRH in turn stimulates the secretion of adrenocorticotropic hormone (ACTH) from corticotroph cells in the anterior pituitary gland. ACTH is released into circulation and induces the secretion of glucocorticoids from the zona fasciculata and zona reticularis of the adrenal gland. Glucocorticoids such as cortisol (cattle) and corticosterone (mice) trigger a multitude of physiological responses throughout the animal. A negative feedback system is in place to regulate the production of glucocorticoids, where high levels of cortisol or corticosterone in blood inhibit the production of ACTH and CRH.

An animal's defense against stressors begins once the central nervous system has been ‘activated' in response to the threat. This can lead to a combination of the following four responses: behavioral, autonomic nervous system, neuroendocrine, and immune responses (37). The most rudimentary response is a behavioral response in which an animal simply attempts to avoid the stressor. Following a behavioral response the autonomic nervous system can be triggered in the context of a “fight or flight” response. This is a short-term response, characterized by elevated levels of circulating catecholamines, epinephrine, and norepinephrine (NE) which affect cardiac, respiratory, muscular, metabolic and other physiological functions of the host (37). The third response is the activation of the hypothalamic-pituitary-adrenal (HPA) axis, and is associated with the elevation of circulating glucocorticoids, steroid derivatives that can have prolonged and substantive effects on the long-term health of the host (Figure 2). Lastly the immune system can activate the innate and adaptive responses. Importantly, both glucocorticoids and catecholamines can influence an immune response, albeit they are temporally distinct from one another, following challenge to a stressor (37).

As a consequence of the stress response, the animal can enter a pre-pathologic or pathologic state. It is in both these stages that energy requirements used to maintain a specific homeostatic function are shifted toward the physiological response associated with exposure to a stressor. This situation can substantively alter homeostatic biological function and cause the induction (pre-pathologic stage) or progression (pathologic stage) of disease (37). This has a detrimental effect on the livestock producer, as energy reserves are redirected from animal performance to coping with the stress induced event.

In livestock production, stressors are mainly grouped into the following three categories: psychologic, physical, and physiologic disturbance to homeostasis (36). Importantly, these three categories are not mutually exclusive and often occur in tandem. Psychologic disturbance to homeostasis is associated with fear, and can be present during periods of social mixing, introduction to new environments, exposure to loud noises, and unfamiliar restraints and equipment. Physical disturbance to homeostasis is that associated with animal injury and disease, extreme environmental temperatures, and periods of hunger, thirst, and fatigue (36). Physiologic disturbance to homeostasis in cattle can be associated with the loss of normal endocrine or neuroendocrine function caused by various conditions, including feed restriction and endocrine disorders (36). Following challenge with an inducer of stress, the host responds with a relatively uniform biological process that counteracts the stressful event in order to return to physiological homeostasis. Briefly, a stressor will stimulate neuroendocrine systems including the HPA axis and the sympathetic nervous system (36). In the context of the HPA axis, diverse stimuli trigger the secretion of corticotrophin-releasing hormone (CRH) and vasopressin (VP) from the hypothalamus, and both of these hormones stimulate the secretion of adrenocorticotropic hormone (ACTH) from corticotroph cells in the anterior pituitary gland (Figure 2). There ACTH is released into circulation and induces secretion of glucocorticoids from the zona fasciculata and zona reticularis of the adrenal gland (36). An increase in glucocorticoid levels in blood will trigger a constellation of physiological responses. These include activating gluconeogenesis processes within the liver, in which different macromolecules, including amino acids and lipids, stimulate synthesis and secretion of catecholamines and modulation of immune system function (36).

Glucocorticoids are comprised of different steroidal molecules. Cortisol is the main glucocorticoid produced in the adrenal cortex of ruminants, while in other species, such as mice, the main glucocorticoid produced by the adrenal gland is corticosterone. Glucocorticoids are lipophilic and can penetrate cells through the lipid plasma membrane. There are two main intracellular glucocorticoid receptors; mineralocorticoid receptor (MR), and glucocorticoid receptor (GR). Corticosteroids have higher affinity for MR than GR; as such, at low basal physiological levels glucocorticoids primarily bind to MR. In contrast, following a stressful event, glucocorticoids can circulate in high quantities which enables binding to GR. Immune cells such as macrophages and T lymphocytes express GR as a primary receptor. It is suggested that this receptor is responsible for immunologic changes that occur in the presence of high levels of glucocorticoids (38). The exact cellular mechanisms on how glucocorticoids alter the immune response are not entirely clear at present. The GR receptor remains inactive within the cytoplasm, but following the binding of glucocorticoids, the receptor translocates into the nucleus and binds to glucocorticoid response elements (GRE) (38). In the nucleus transcription of immune elements can be modulated via a number of proposed mechanisms. One model suggests that GR recognizes a putative hormone response element in the sequence of diverse cytokines and this enhances or represses transcription of various genes. This mechanism does not appear to be involved in the expression of important cytokines associated with immune function given that not all cytokines possess this response element (38). The most accepted mechanism is the down regulation of NF-κβ following GR translocation to the nucleus. It is believed that GR can activate the transcription of an NF-κβ inhibitor (IKb alpha), and the inhibitor will sequester NF-κβ in the cytoplasm and prevent it from entering the nucleus. Another proposed mechanism is the direct binding of GR to NF-κβ causing its inhibition (38). Regardless of the mechanism, glucocorticoids are potent immunomodulators able to reduce expression of important pro-inflammatory cytokines such as TNF-α, interleukin 1 (IL-1), and interleukin 12 (IL-12), or inhibiting NF-κβ and interleukin 6 (IL-6), as well as decreasing T and B lymphocytes numbers in the host (14). Thus, it is plausible that during periods of prolonged disturbance to homeostasis immune function is altered in cattle, which subsequently affects the microbiota in the intestinal tract, thereby opening niches or possibilities for invading bacteria that are not able to survive or colonize under normal circumstances.

Cortisol concentrations in plasma of cattle are used as a measure of HPA axis activation (39). It is secreted in a pulsatile manner, and as such, the secretion of cortisol follows a diurnal cycle. Cattle show increases in cortisol release in response to acute periods of stress that include dehorning (40), restraint, or mixing with unknown animals (41). The release of cortisol is a relatively slow process, requiring a few minutes after the stressful event to reach peak levels in blood. Basal levels of cortisol in cattle are usually <15 nmol/L but can increase to 60–200 nmol/L, in response to a stressor (41). During periods of chronic disturbances to homeostasis, the levels of cortisol are lower than levels associated with acute responses. Even when cortisol is at basal levels under chronic stress, the activation of the HPA system can still be observed.

Similar to cortisol, under situations of alterations to homeostasis the adrenal medulla, composed primarily of chromaffin cells, will also produce catecholamines, epinephrine, NE, and dopamine (24). These hormones are first synthesized from L-dopa into dopamine, and later into NE and epinephrine. Norepinephrine and dopamine are located in sympathetic terminal nerve endings throughout the nervous system, including the enteric nervous system (24). Epinephrine is secreted in the central nervous system and the adrenal gland, and can reach the intestinal tract through the systemic circulation system. Catecholamines prepare the body for an attack or flight response, and can increase heart rate, constrict blood vessels, dilate bronchioles, and increase metabolism (36). When secreted over prolonged periods catecholamines can also alter immune function. Immune cells, such as macrophages and T lymphocytes, express β2 adrenergic receptors which can modulate immune responses (38).

## Quorum Sensing and Impacts of Host Stress

**Figure 3:**
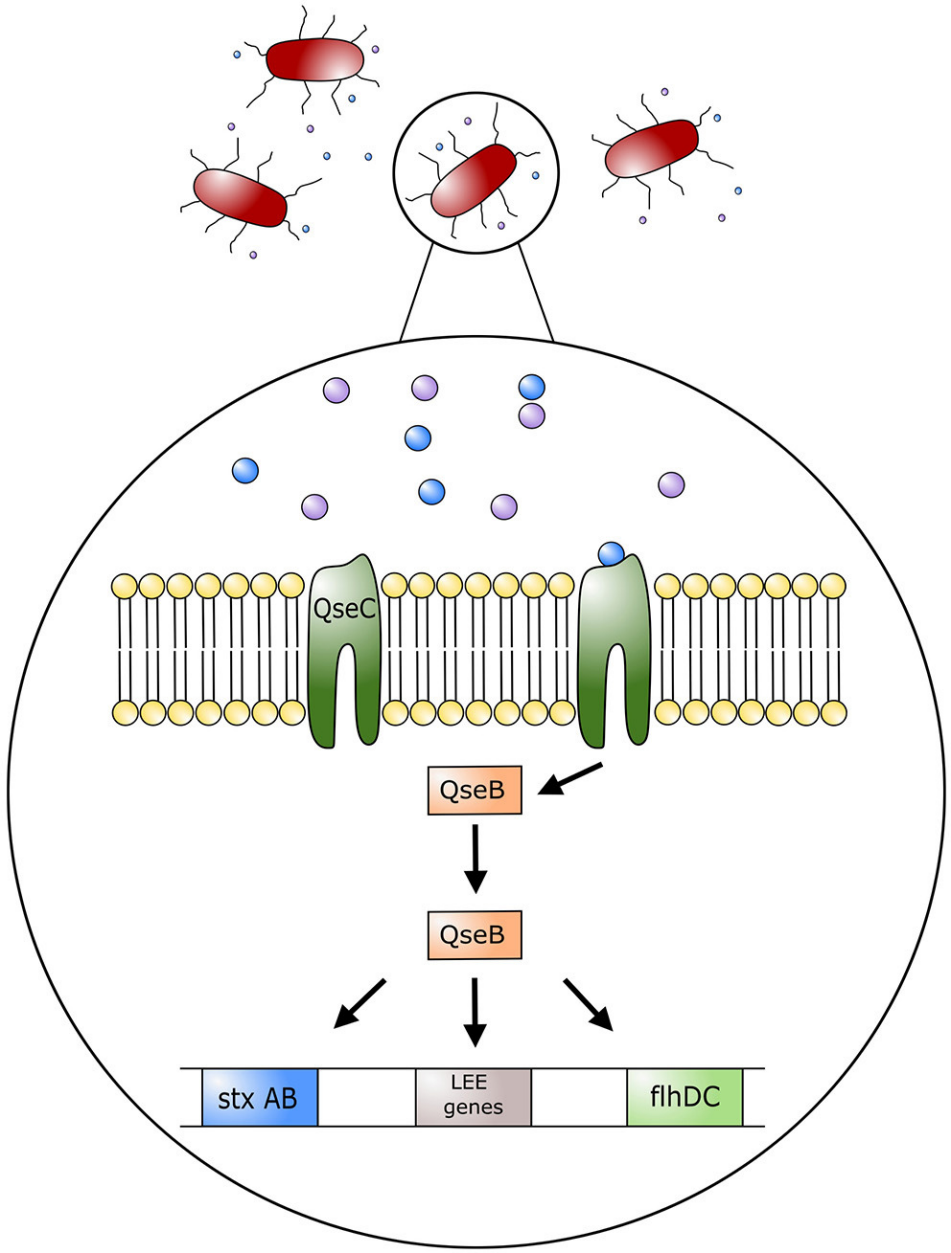
Model of regulation of *Escherichia coli* O157:H7 pathogenic genes by AI-3, epinephrine and norepinephrine. Quorum sensing molecule AI-3, and host stress hormones epinephrine and norepinephrine bind to the bacterium membrane receptor (QseC); this in turn triggers the phosphorylation of the response regulator QseB, which stimulates the expression of the locus of enterocyte effacement (LEE) genes, motility genes (flhDC), and Shiga toxin (StxAB). LEE genes will encode for the type three secretion system (TTSS) and promote attaching effacing lesions on host cell membranes.

The impact of stress on colonization of bacteria in the GIT and the induction of EHEC associated disease has not been fully investigated. Studies examining inter-bacterial signaling and bacterial-host signaling have been conducted, and provide interesting information on the interaction of stress hormones and intestinal bacteria (15, 42, 43). Bacterial interspecies communication can take place via cell-to-cell signaling by a mechanism called quorum sensing. *Escherichia coli* are able to produce molecules that bind to surface receptors of other *E. coli* cells, thereby stimulating or inhibiting a response. As an example, *E. coli* O157:H7 produces the autoinducer 3 (AI-3), a molecule that binds to a histidine kinase membrane receptor leading to the activation of virulence inducing transcription factors. This quorum sensing system is composed of quorum sensing regulators, named Quorum sensing *E. coli* (Qse), that will either act as histidine kinase membrane receptors or transcription factors that can regulate expression of virulence factors (24, 44) (Figure 3). Enterohemorrhagic *E. coli* has the histidine kinase membrane receptor QseC that specifically recognizes quorum sensing molecule AI-3. Once activated, QseC will phosphorylate QseB, a response regulator that promotes the activation of LEE genes encoding for the TTSS as well as motility virulence genes activating flagella (24). Furthermore, QseB activates production of a second receptor QseE, another quorum sensing membrane receptor that promotes A/E lesion formation (24, 44). By signaling with commensal *E. coli* and other enteric bacteria, *E. coli* O157:H7 can activate genes responsible for colonization of the intestinal tract (43). Importantly, this communication system can alert EHEC when it has reached the large intestine, given that commensal bacteria such as *E. coli, Enterococcus, Clostridium*, and *Bacteroides* spp. also produce AI molecules as part of their communication system (43). Communication between these commensal bacteria and EHEC will help direct EHEC to its location in the intestinal tract as well as induce activation of its virulence factors (43).

Histidine kinase receptors in EHEC, such as QseC, have been found to have a key role in communication between the mammalian host and bacteria. Such an interaction is known as inter-kingdom signaling. As such, the host catecholamines, epinephrine and NE, act as agonists on QseC, the same receptor used by quorum sensing molecule AI-3 (44). In this manner host stress molecules can stimulate the activation of EHEC virulence factors such as TTSS and flagella (43). In essence, AI-3 cross talks with epinephrine and NE. Furthermore, by sensing catecholamines, EHEC can recognize an altered physiologic and immunologic function in the host (44). As an example, recognition of catecholamines by QseC results in the transcription of flagella genes and TTSS, to facilitate colonization by EHEC. In the presence of epinephrine and NE, attachment of EHEC to HeLa cells was increased, as was its motility and ability to form biofilms (42). Furthermore, injections of NE into bovine ligated ileal loops showed increased epithelial cell adherence and induction of enteritis by EHEC (15). In this regard, there were increased neutrophilic infiltrates within the lamina propria, submucosa, and intestinal lumen. In addition, extensive A/E lesions were found in ileal loops inoculated with EHEC in the presence of NE (15), and no EHEC incited lesions were found in the loops only inoculated with a diluent (15). Collectively, these observations suggest that stress in cattle can influence the colonization of EHEC within the intestinal tract through inter-kingdom quorum signaling, thereby promoting its virulence to facilitate survival.

## Nutritional Basis for Intestinal Colonization

Freter et al. (45) highlighted that to colonize and survive within the GIT an organism must successfully use at least one limiting nutrient more efficiently than other competing bacteria. In this manner, an organism is defined by its ability to occupy a nutrient defined ecological niche that differs from the other species present. The population size of a particular bacterium can be defined by the amount and availability of the nutrients required to survive. Based on these principles, many studies have tried to determine the complex catabolic mechanisms needed to metabolize nutrients from intestinal tract mucus by EHEC. The nutrients accessed from intestinal tract mucus by EHEC and its commensal counterpart have been studied in both cattle and mice (16–18, 46).

Mucus is an important component of the mucosal barrier and assists in protecting the host from pathogen invasion. Mucus can also be used as a substrate for bacterial growth, and it is a good source of energy for carbohydrate metabolism. Fabich et al. (16) compared *in vitro* EHEC and commensal *E. coli* metabolism of intestinal carbohydrates normally present in the mucus of mice. Glycoproteins, which form mucus, found in the murine intestinal tract are comprised of 13 different monosaccharides which are potentially available to EHEC via degradation by anaerobic enteric bacteria (16). Notably, EHEC is unable to hydrolase these glycoproteins by itself, as it does not possess the necessary hydrolases (16). Enterohemorrhagic *E. coli* grown in mucus express catabolic pathways for the utilization of seven of the thirteen monosaccharides. These carbohydrate metabolic mechanisms have been confirmed in a human isolate of EHEC (EDL933), but differed from that of a commensal human *E. coli* isolate (MG1655) (16). The differences observed in carbohydrate utilization suggest that both pathogenic and non-pathogenic *E. coli* can coexist in the intestine by occupying unique niches, and that more than one commensal strain is necessary to cover the broad range of carbohydrate metabolic pathways that EHEC can exploit (16). In this regard, multiple *E. coli* commensal strains that covered the full spectrum of carbohydrates used by EHEC were needed to competitively exclude EHEC (17). This is supported by the observation that multiple commensal *E. coli* organisms can co-colonize and coexist in the intestine of streptomycin-treated mice based on differences in the types of carbohydrates that these bacteria need for colonization (17).

In cattle, the main fermentable saccharides from mucus in the small intestine are galactose, N-acetylglucosamine (NAG), N-acetylgalactosamine (GalNAc), fucose, mannose, and *N*-acetyl neuraminic acid (46). The human EHEC isolate, EDL933, is able to utilize all six sugars, and competition assays suggest that the capacity of EHEC to metabolize mannose, NAG, GalNAc, and galactose is critical to achieve maximum growth of the bacterium within the bovine intestine. The genes required to utilize the six sugars are expressed at maximal levels during the exponential growth phase, except for the gene required to degrade mannose, which has the highest expression during stationary phase (46). Bertin et al. (46) established that mannose and NAG catabolism provides EHEC with the greatest competitive growth advantage in cattle, and that this differs from the sugars needed for colonization of the mouse intestine.

Although the nutritional environment is only one factor involved in colonization resistance (i.e., the mechanisms by which the autochthonous microbiota regulates pathogens), the determination of each bacterial nutritional requirement is important to understand the mechanisms of competitive exclusion between non-pathogenic and pathogenic *E. coli* strains. It is noteworthy that *E. coli* O157:H7 and commensal *E. coli* can metabolize multiple carbohydrates in order to survive in the intestinal tract, rendering the understanding of mechanisms involved in the competition for nutrients even more challenging.

## Virulence Gene Expression and Influence of the Metabolic Landscape

Commensal bacteria can alter the nutritional environment of EHEC as well as influence the metabolic landscape of the bacterium in the GIT. *Escherichia coli* O157:H7 can react to subtle changes in the environment to either activate or suppress the expression of virulence factors (47, 48). Changes in carbohydrate concentrations can have an impact on gene expression in EHEC. For example, growth under glycolytic conditions (environments rich in glucose, such as the duodenum and jejunum) can inhibit the expression of the transcription factor *Ler*, a LEE-1 encoded regulator that controls the transcription of LEE operons (48). Conversely, growth in a gluconeogenic environment (an environment with low glucose concentrations, such as the distal colon) can activate the expression of LEE operons and consequently the virulence of EHEC (48). This demonstrates that *E. coli* O157:H7 can recognize and follow a gradient of nutrient concentrations, repressing expression of factors involved in colonization during unfavorable conditions (i.e., within the small intestine) and activating these factors under the favorable nutritional conditions (i.e., within the large intestine) (49). *Escherichia coli* O157:H7 transcription factor *Cra* can also sense fluctuations in sugar concentrations within the GIT and thus activate expression of LEE genes. The carbohydrate content present within this environment can be altered by the presence of other bacteria, and in this manner can stimulate or inhibit the production of virulence factors by EHEC, thus impacting intestinal colonization and survival (47). *Bacteroides thetaiotaomicron* increased the expression of 20% of the *E. coli* genome (47). The presence of *B. thetaiotaomicron* augmented the expression of LEE genes, *Stx2a*, and the *StcE* gene, a gene that encodes a mucinase. Furthermore, *B. thetaiotaomicron* increased expression of *Ler* and TTSS structural proteins such as *EspA*, and TTSS receptor Tir (47). In summary, modification of the local GIT environment by other bacteria can influence the expression of virulence factors in EHEC, and thereby modulate growth and colonization of EHEC within the intestinal tract.

## Mouse Models to Evaluate Intestinal Colonization

There are many advantages of using mice as a model to look at bacterial colonization mechanisms. It must be stressed that studies using mice are by no means a replacement for utilizing ruminants as models to study *E. coli* O157:H7. It is merely a first step in attempting to understand basic bacterial mechanisms that can later be tested in ruminants. The advantage of using mice is clear from an economical perspective. Mice have low maintenance costs, are small, and have short generation times which allows for fast and agile research experiments. Additionally, the complete genome of the mouse is well characterized, which allows for manipulation of the genome. Consequently, different varieties of genetically engineered mice exist, including both knock-in and knock-out genetic models. The use of recombinant models facilitates research to elucidate mechanisms.

The predominant animal model used to study pathogenesis of *E. coli* O157:H7 in humans is the mouse. The main murine models utilized are conventional mice treated with streptomycin to induce an intestinal dysbiosis or germ-free mice (50). Germ-free mice are devoid of microorganisms (51). In contrast, mice with a fully established and known microbiota are considered gnotobiotic. Thus, germ-free mice colonized with known strains of bacteria will become gnotobiotic mice (52). Conventional mice (mice with undetermined normal flora) have also been used as *E. coli* O157:H7 colonization models. The fidelity of this model, however, can be compromised by the presence of resident commensal *E. coli* and other enteric bacteria that potentially confound EHEC colonization studies (53, 54). Long-term fecal shedding was achieved in only one of the conventional mice tested, and intestinal colonization rates of *E. coli* O157:H7 were low (53, 54). Sheng et al. (35) inoculated conventional mice with human and bovine *E. coli* O157:H7, and tested bacteriophage action against the bacteria in the gastrointestinal tract. However, other models have proven better to study EHEC induced intestinal disease. Streptomycin-treated mice have been used extensively as a human model to study EHEC infection, colonization, and competitive exclusion (16–18, 55–57). Treatment with streptomycin induces intestinal dysbiosis by inhibiting the growth of commensal facultative anaerobic bacteria. More specifically, bacterial densities of enterococci, streptococci, lactobacilli, anaerobic lactobacilli, and bifidobacteria are reduced, while *Bacteroides* and *Eubacterium* spp. remain unaffected by antibiotic treatment (56). This allows an opportunity for EHEC and other commensal *E. coli* to successfully colonize and persist within the mouse intestinal tract. Germ-free mice are another model that has been used to study EHEC colonization and infection. No competition occurs between EHEC and resident bacteria as the GIT lacks microorganisms. Several studies have employed this model. Takahashi et al. (20) observed robust colonization of a hypertoxigenic EHEC strain (10^8^-10^9^ CFU/g of feces) by day 6 post-inoculation, and increased death in mice mono-associated with EHEC on day 7 post-treatment. Taguchi et al. (19) also observed high colonization rates of EHEC in the intestinal tract of germ-free mice with a corresponding manifestation of colonic injury and inflammation. Other studies have also observed successful EHEC colonization in germ-free mice, with indications of disease that include animals with marked neutrophilic necrotic enteritis that on occasion succumbed to disease (58). Moreover, Eaton et al. (21) tested ten different EHEC serotypes in Swiss-Webster germ-free mice, and showed colonization of all bacterial strains within the GIT. Colonization was unaffected by the dose or time interval of the oral inoculation, and bacterial shedding was robust at 10^9^ to 10^12^ CFU/g of feces. This demonstrates that regardless of the inoculation dose, EHEC will colonize at a similar final density. Eaton et al. (21) claimed that germ-free mice are exquisitely susceptible to colonization by EHEC and that inoculation with numbers as low as 100 cells can increase growth to a persistent intestinal bacterial density of 10^9^ CFU/g or more within a single day. They also observed that EHEC colonizing the intestinal tract of germ-free mice caused clinical symptoms of disease, including lethargy, dehydration, polyuria, and polydipsia, with death of challenged mice occurring 4 to 7 days post-inoculation. Interestingly, inoculated mice did not develop diarrhea, but cecal edema was observed (21). As well, when inoculated with ten different EHEC strains only seven strains caused disease in mice, and it was speculated that the most significant cause of disease was renal injury; similar to HUS in people (21).

Swiss Webster germ-free mice inoculated with 10^6^ CFU of EHEC have been used in a number of studies (59, 60). Mice inoculated with strain EDL933 became moribund and exhibited lower body weights, renal tubular necrosis, and renal failure 3 weeks post-inoculation. The same bacterial strain, but lacking the ability to produce Stx, did not develop disease and mice exhibited normal renal morphology (60).

In summary, germ-free and gnotobiotic mice are valuable models to study colonization of the intestinal tract by EHEC. In this regard, studies can be directed at elucidating the competition of inoculated bacteria for the same niche in a highly prescribed manner (i.e., with no or limited confoundment from resident enteric bacteria). Although the streptomycin induced dysbiosis model allows for colonization of the murine intestinal tract by *E. coli* O157:H7, the presence of a resident bacterial flora potentially limits the elucidation of specific bacterial colonization mechanisms.

## Location of Intestinal Colonization in Mice

Commensal *E. coli* (HS, MG1655 and Nissle 1917 strains) can be isolated from mucus of the entire intestinal tract of streptomycin treated mice with the highest densities collected from cecal and colonic mucus (56). In the absence of these commensal *E. coli* bacteria, *E. coli* O157:H7 effectively colonized the entire GIT (56). Similar to cattle, the highest densities of commensal *E. coli* were present in feces of mice as compared to bacteria isolated directly from intestinal samples (56). In another study, densities of commensal *E. coli* and EHEC were ten-fold higher in cecal and colonic mucus as compared to the rest of the intestinal tract (18). Although both strains (commensal and pathogenic) were found in mucus along the entire intestinal tract, higher densities of cells were observed in the large intestine. Using fluorescence *in situ* hybridization (FISH), *E. coli* O157:H7 (EDL933) was associated with the epithelium and mucus along the intestinal tract, with numbers ten-fold higher in the large intestine (18). Surprisingly, EHEC failed to grow in cecal luminal content and in contrast to EHEC, commensal *E. coli* were not associated with the epithelium (18). Finally, in germ-free mice challenged with EHEC, Eaton et al. (21) observed that EHEC colonized the entire intestinal tract, with the highest densities of the bacteria in digesta from the cecum and colon, as well as from the ileum. High levels of bacterial adherence to the epithelium were also observed in the ileum and cecum, with lower levels of adherence observed in the colon. Furthermore, densities of bacteria within digesta were higher throughout the intestinal tract rather than in association with the epithelium (21).

## Mechanisms of Competitive Exclusion in the Intestinal Tract

In a healthy GIT, a mutually beneficial relationship exists between the microbiota and the host. The host provides ‘commensal' bacteria with a stable growth environment and nutrient supply, and in return, the commensal microbiota help develop and modulate the immune system, provide nutrients, and assist with both the prevention of colonization and elimination of pathogens from the GIT (61). The mechanisms by which commensal bacteria inhibit pathogen colonization within the intestinal tract is known as colonization resistance (CR) (61, 62). There are several mechanisms that lead to successful CR of pathogens within the GIT. These mechanisms include; direct inhibition of pathogens, nutrient depletion in specific intestinal locations, and modulation of intestinal and extra-intestinal immune responses (13, 62). The production of antimicrobial peptides, such as bacteriocins, the generation of inhibitory metabolites (e.g., butyrate and acetate), competition for binding sites, and stimulation of mucus secretion are all processes involved in direct inhibition (13, 62).

Many nutrients required for bacterial growth are limited within the GIT, and the ability of bacteria to access and assimilate nutrients is vital for their growth. The high diversity of the microbiota within the intestinal tract of mammals results in vigorous competition for limited nutrients between all microorganisms (13, 63). In this regard, if a bacterial pathogen is unsuccessful at accessing required nutrients (e.g., as a result of competition by autochthonous bacteria) it is unable to successfully colonize the intestinal tract at densities needed to infect the host and subsequently incite disease. In some situations, pathogens can benefit from the presence of inflamed tissue within the intestine by possessing adaptive systems that preferentially overcome acute or chronic inflammation while other commensal organisms are eliminated by the inflammatory processes (62, 64). Some pathogens have even evolved mechanisms that stimulate a pro-inflammatory immune response that reduces the diversity of commensal bacteria at the site of inflammation, allowing the pathogen to occupy niches that would have been previously unavailable (64). Moreover, pathogenic microorganisms have acquired the ability to differentially exploit niches within the intestinal tract. For example, many pathogenic bacteria such as EHEC possess specific pathogenicity factors such as adhesins or invasins that aid in epithelial attachment and enable the pathogen to successfully colonize the GIT. Other bacteria can breach the mucus barrier, including the tightly adherent mucus layer, avoiding entrapment of the bacteria within the mucus (65, 66). Finally, certain *Bacteroides* spp. possess a modified LPS that is less immunogenic as compared to the highly immunogenic LPS of EHEC, thus reducing host recognition of the bacteria (65).

## Competitive Exclusion in Cattle

Several observational competitive exclusion studies in cattle using probiotic *E. coli* as a strategy to eliminate EHEC from the GIT have been explored (67–69). In some instances, shedding of *E. coli* O157:H7 in adult cattle and calves was reduced following challenge with non-pathogenic microorganisms. Notably, colicin E7-secreting *E. coli* reduced EHEC numbers in cattle (67). Colicins are antimicrobial proteins produced by some *E. coli* strains that can eliminate other bacteria by inhibiting peptidoclycan synthesis, forming membrane pores or cleaving DNA (67). However, given the high diversity of the microbial community within the intestinal tract of cattle it is hard to conclude if the reduction of EHEC was associated with colicin E7 or due to other mechanisms. Conducting experiments directly in cattle that investigate mechanisms of competitive exclusion presents a number of salient limitations. These limitations can be related to animal husbandry practices, such as cost, traction and size of the animals, and genetic, physiological, and microbial heterogeneity.

## Competitive Exclusion in Mice

To date, EHEC competitive exclusion studies performed in mice have mainly focused on utilizing the mouse as a model of human intestinal competition. These studies have been conducted with human *E. coli* O157:H7 isolates and have used human isolates of commensal *E. coli* as competitive strains (16, 18, 55, 56). Competition between bovine *E. coli* O157:H7 and bovine commensal *E. coli* isolates have not been utilized in a mouse model. In this manner, the mouse model has not been used to study these factors in bovids, where the progression of intestinal inflammation mimics intestinal changes within the bovine host (i.e., a colonization model of chronic inflammation) without development of kidney injury and renal failure.

Competitive exclusion studies conducted in mice have provided valuable information on the potential of excluding EHEC from the intestine. The specific mechanisms involved in these competitions, however, are complex and are not fully elucidated. Presently, mechanistic studies that have been conducted have mainly focused on the competition for limiting nutrients between commensal *E. coli* and EHEC, and in particular, researchers have selected non-pathogenic *E. coli* which metabolize all nutrients needed by EHEC to achieve colonization resistance (17). Moreover, researchers analyzing different carbohydrates metabolized by both commensal *E. coli* (MG1655) and EHEC (EDL933) strains concluded that both bacteria can coexist and colonize the GIT of mice. In addition, despite requiring some of the same carbohydrates to survive, both bacteria were able to co-colonize based on their ability to metabolize different sugars (16). The diverse metabolic tools that *E. coli* O157:H7 possesses and the ability of different *E. coli* strains to co-exist suggests that a single commensal *E. coli* strain will likely be insufficient to outcompete EHEC for colonization within the intestine. Leatham et al. (56) showed that individually, MG 1655, HS and Nissle 1917 *E. coli* strains were incapable of outcompeting EDL 933 EHEC strain for GIT colonization in streptomycin-treated mice. However, when co-administered, these three commensal bacteria, having different nutrient requirements, were able to accomplish a four-fold reduction in the number of EHEC collected in feces. Importantly, these commensal bacteria were introduced to the mice 10 days prior to the challenge with EHEC, allowing for successful colonization of the commensal microorganisms, and thus preventing EHEC growth in the GIT. Bacteria previously established in the intestinal tract (i.e., occupying specific niches) have a competitive advantage over the bacteria that are newly introduced and require the same niche. Notably, the specific mechanisms for exclusion of EHEC by the three commensal *E. coli* above were not determined. Nutrition, innate immunity, or the indigenous microbiota (i.e., since the mice used were administered streptomycin to incite a dysbiosis, an undefined microbiota remained) could all have been involved in the competition. Contrary to this study, Gamage et al. (55) competed EHEC with a single commensal *E. coli* isolate in streptomycin-treated mice and showed reduced EHEC concentrations in feces at 4 days post-inoculation. The mechanisms behind this reduction were undetermined.

## Defined Microbiota Mice

Germ-free mice are considered ‘free of demonstrable viable microbial associates', and they are a valuable model for studying inter-bacterial interactions and host-bacterial interactions *in vivo* (70). The administration of known bacterial taxa into the intestinal tract of germ-free mice (i.e., gnotobiotic) allows researchers to specifically focus on the introduced bacteria without the confounding effects of the enteric commensal bacterial community. The absence of a diverse commensal microbiota using gnotobiotic mouse models allows researchers to study specific interactions in a logistically feasible manner, and to formulate hypotheses in an informed manner for subsequent testing in agnotobiotic models. Notwithstanding the value of germ-free and gnotobiotic models, it is important that researchers recognize the limitations of these models. In this regard, the small intestine, extra-intestinal tissue such as lymph nodes, and on occasion the liver of germ-free mice have reduced weights in comparison to conventional mice. In contrast, tissues that are naturally not in contact with microorganisms, such as extra-intestinal organs and the nervous system are equivalent in weight and size to conventional mice (70). Moreover, immunogloblulin A (IgA) and immunogloblulin G (IgG) are produced in smaller quantities following antigenic stimulation in germ-free mice. The structure of the intestinal tract in germ-free mice also differs substantially from conventional mice and it has been shown that bacteria will affect normal development (71). The small intestine is thinner and hypocellular with fewer numbers of lamina propria macrophages and lymphocytes. The villus crypts are shallower, with lower germinative cell mitotic activity and Peyer's patches are reduced in size (71). Notably, the intestinal lymphoid tissue in germ-free mice is still functional and capable of mounting a response to antigenic stimulation (71). One prominent feature of the intestinal tract of germ-free mice is the enlarged cecum, which can weight up to ten times more and contain six times more cecal content as compared to conventional mice. The cecal content also has a more liquid consistency and a hypotonic osmolarity. Finally, motility and peristaltic waves of the intestinal tract are significantly reduced as compared to mice with an established intestinal microbiota (70, 71).

## Mice to Elucidate the Influence of Host Stress

As indicated previously, a stressful event can trigger the activation of the HPA axis elevating glucocorticoids in blood and thereby induce metabolic changes within the host. Mice have been used as animal models to study stress, depression, and anxiety. These models can be achieved by either directly administering exogenous corticosterone to a mouse or by placing the mouse under stressful conditions and stimuli, such as physical restraint or forced periods of swimming, ultimately elevating endogenous corticosterone blood levels (72).

The administration of glucocorticoids such as corticosterone to induce physiological changes representing chronic stress has been employed through various means including administration in drinking water, subcutaneous injections, oral gavage, and the implantation of slow-release subcutaneous pellets (72). Corticosterone in drinking water is the most commonly used method, although it can be difficult to standardize dosage due to different amounts of water ingested by the animals. However, this method is preferred and often used given its ease of administration, especially when using germ-free or gnotobiotic mice, as this method of administration reduces the potential of accidental bacterial contamination of the mice (73–76). Another advantage of providing corticosterone in water is the reduction in stress associated with animal handling and drug administration (73). Indeed, injections of corticosterone or subcutaneous surgeries required to implant corticosterone pellets can be particularly traumatic for mice, potentially generating unwanted stress and spiking endogenous corticosterone levels in control animals. Although dosage standardization is superior with corticosterone pellets or injections, inadvertent stress and the risk of bacterial contamination of germ-free or gnotobiotic mice are paramount considerations, and both are reduced by administering corticosterone *per os* in drinking water.

Multiple studies have examined the impact of different dosages of corticosterone on the induction of stress responses in mice. In this regard, a concentration of 25 μg/ml corticosterone in a 1% ethanol solution (i.e., low dose) will induce modest changes in the physiology and behavior, while a high dose of 100 μg/ml corticosterone in a 1% ethanol vehicle causes more prominent effects (74, 75). Importantly, it has been shown that corticosterone-induced physiological stress in mice also corresponds to behavioral changes (77). Examples of behavioral modifications include increased levels of depression-like and anxiety-like behaviors in corticosterone treated mice. Behavioral changes are evaluated using different tests and some of these include open field exploration test, forced swim, elevated plus maze challenge (tests the anxiety-like behavior of mice by observing the movement, entries and exits of mice between an area protected by walls and an open unprotected area), tail suspension, and others (77).

## Conclusions

*Escherichia coli* O157:H7 has been studied extensively. However, there are many aspects of the host-pathogen interaction that still remain unknown, particularly in relation to the interaction in ruminants. Current methods to control *E. coli* O157:H7 in cattle with the goal of preventing transmission of the pathogen to people have largely been unsuccessful. Commensal bacteria have been found to influence the course of EHEC's colonization (47), but to date, competitive exclusion studies utilizing mice have mainly focused on excluding EHEC from the perspective of human medicine, and have thus used human commensal *E. coli* to study competition, colonization, pathogenesis, and disease (16, 18, 55, 56). The ability of bovine commensal *E. coli* isolates to exclude EHEC has not been examined, nor have mice models been used for EHEC colonization studies that simulate the bacterial interactions encountered in cattle. Moreover, the mechanisms involved in the interaction mentioned above are currently unknown, and the acquisition of such information could be key toward the development of innovations to mitigate this important zoonotic pathogen on the farm.

The immune response generated by cattle and mice colonized by *E. coli* O157:H7 over prolonged periods, and the impact of the host immune system on the bacteria are not fully understood. Moreover, the presence of the enteric microbiota makes assessing changes to colonization even more challenging. As such, there can be value in using murine models colonized with an *E. coli* O157:H7 isolated from cattle to investigate colonization mechanisms and bacteria host interactions. Streptomycin-treated mice can be a useful model to observe *E. coli* O157:H7 impacts on the GIT commensal community. Additionally, the lack of microorganisms in the GIT of germ-free mice is an advantage for highly prescribed studies in which the goal is to elucidate mechanistic interactions between the host and *E. coli* O157:H7. To date, most studies in mice have focused on determining the effects of Stx on the host, mainly from a perspective of studying the pathogenesis of HUS. Relatively few studies have examined interactions between *E. coli* O157:H7, commensal bacteria, and commensal *E. coli* within the intestinal tract. A potentially interesting approach would be the use of a bovine flora murine model to validate findings observed in gnotobiotic mice. Human flora mouse models have been established (78), and the establishment of a bovine colonic microbiota in gnotobiotic mice to emulate the distal colon environment of cattle is achievable. Competition between bovine *E. coli* O157:H7 and autochthonous commensal bacteria could be studied in this model in a cost effective manner.

It has been proposed that EHEC resides in the lymphoid follicle rich mucosa of the terminal rectum of cattle (9); however, knowledge on mechanisms of colonization and EHEC interaction with the host are still broadly undefined and their elucidation could potentially provide valuable information toward comprehending EHEC's behavior in the GIT. Furthermore, it is not fully resolved as to why EHEC colonization of the intestinal tract of adolescent and adult cattle does not incite clinical disease. This characteristic suggests ruminants act as a silent reservoir creating a complex scenario for both detecting and reducing EHEC in cattle operations. Moreover, EHEC environmental shedding is not fully understood. Shedding patterns have been shown to be variable throughout the seasons of the year, presenting short peaks of shedding followed by prolonged periods of intermittent shedding of low numbers of bacterial cells, or none at all (28, 29). Additionally, the immune response mounted toward *E. coli* O157:H7 in the large intestine needs further exploration in order to enhance potential mitigation strategies. Little is known about the cellular and humoral responses in the intestinal tract when *E. coli* O157:H7 is forced to compete for colonization niches with commensal bacteria, particularly commensal *E. coli* strains.

The impact of stress on *E. coli* O157:H7 intestinal tract colonization and disease has received relatively limited attention (43). The influence of stress molecules, such as catecholamines, on *E. coli* O157:H7 has been studied in cell cultures and ileal loops; however, there is little knowledge on the impact of stress on *E. coli* O157:H7 colonization *in vivo*. Furthermore, corticosterone, a hormone elevated for prolonged periods during chronic stress, has not been studied as an inducer of stress in an *in vivo* model of *E. coli* O157:H7 colonization. Additionally, there is little information on the impact of stress on EHEC intestinal colonization, intestinal immune function, and competitive exclusion between commensal of *E. coli* and *E. coli* O157:H7 in a gnotobiotic mouse model.

## Author Contributions

MEL and GDI conceived and designed the review, and wrote the manuscript. MEL prepared figures. RREU contributed content to and reviewed the final version of the manuscript. All authors contributed to the article and approved the submitted version.

## Funding

The review was funded by a grant from Agriculture and Agri-Food Canada (Peer Review Project #1097).

## Conflict of Interest

The authors declare that the research was conducted in the absence of any commercial or financial relationships that could be construed as a potential conflict of interest.

## Publisher's Note

All claims expressed in this article are solely those of the authors and do not necessarily represent those of their affiliated organizations, or those of the publisher, the editors and the reviewers. Any product that may be evaluated in this article, or claim that may be made by its manufacturer, is not guaranteed or endorsed by the publisher.
